# Mapping Protein-Protein Interactions With Combinatorial Peptides

**DOI:** 10.1002/cfg.100

**Published:** 2001-10

**Authors:** Brian K. Kay

**Affiliations:** Department of Pharmacology 1300 University Ave. University of Wisconsin-Madison Madison WI 53706-1532 USA


**Comparative and Functional Genomics**
*Comp Funct Genom* 2001; **2**: 304–306.
**DOI:** 10.1002/cfg.100

**Conference Review**

### Mapping protein-protein interactions with combinatorial peptides

A presentation for the ESF workshop 'Proteomics: Focus on protein interactions'

Brian K. Kay*
*Department of Pharmacology, University of Wisconsin-Madison, USA*

*Correspondence to:
B. K. Kay, Department of Pharmacology, 1300 University Ave., University of Wisconsin-Madison Madison, WI 53706-1532, USA.
E-mail: bkkay@facstaff.wisc.edu*

Received: 10 July 2001
Accepted: 27 July 2001

**Keywords:** **proteome; phage-display; combinatorial peptides; protein-protein interactions**

Now that a number of genomes have been sequenced, attention has turned to understanding the complement of proteins encoded by the genome, which has been termed the 'proteome'. One important aspect of analysis of the proteome is the identification of which proteins interact with each other; this information is invaluable in surmising the function of each protein and inferring what cellular pathway the protein may be a component of. While a variety of methodologies is widely used to map protein-protein interactions, such as affinity purification followed by mass spectrometry and yeast two-hybrid screening (both of which were covered at this meeting), an alternative approach is the use of phage-displayed combinatorial peptides.

In phage-display, short oligonucleotides are inserted within a gene encoding a capsid (coat) protein of a bacteriophage, so that each viral particle displays a different peptide sequence. While it has been possible to clone and express short peptides attached to each of the five different capsid proteins of bacteriophage M13, the protein products of gene III and VIII are popular cloning sites for expression and display. Several excellent reviews of phage-display can be found elsewhere [6,22,5]. Over the past ten years, a considerable number of phage-displayed combinatorial peptide libraries have been generated [21].

With a library of recombinant phage particles in hand, it is possible to screen them by affinity selection with a variety of protein targets. Three rounds of selection (binding, elution, propagation) are typically sufficient to screen billions of different phage-displayed combinatorial peptides for those that bind to target proteins of interest. While there have been many recent publications of successful selection experiments, some of the more notable examples are the selection of peptide ligands which bind to receptors for erythropoietin [30], N-methyl D-aspartate (NMDA) [16], thrombopoietin [7], fibroblast growth factor [3], and estrogen [17]. When peptides are chemically synthesized corresponding to what is displayed by the selected phage, they generally bind with dissociation constant ($K_d$) values of 5 micromolar to 10 nanomolar to their cognate receptor, and typically have agonist or antagonist activities. Many different types of targets will yield phage after affinity selection, such as enzymes [12], growth factors [10,4], nucleic acids [2], and cells [20,13].

Copyright © 2001 John Wiley & Sons, Ltd.

Proteins involved in eukaryotic signal transduction, cellular differentiation, and apoptosis have been used in phage-display selection experiments. Because of their central roles in cell physiology, understanding the function of these proteins has included mapping the proteins that they interact with in the cell. As was described at the recent ESF Workshop in Rome, one can select peptide ligands for such targets from phage-displayed combinatorial peptide libraries and then use the consensus among the selected peptides to predict what the target protein might bind in the cell. Surprisingly, the consensus often resembles a primary structure within the natural interacting partner of the protein; we have termed this phenomenon 'convergent evolution' [14]. Thus, a fruitful approach for mapping protein-protein interactions is to isolate peptide ligands to a target protein and then identify candidate interacting proteins in a sequenced genome by computer analysis.

Several examples were presented at the meeting. One example involved the two Eps15 Homology (EH) domains of the adaptor protein, intersectin [31], which is involved in endocytosis [18,25] and signal transduction [1,28]. Affinity selection of peptides that bound to these two domains identified the motif, Asp-Pro-Phe (NPF), as their optimal ligands [31], much like other EH domains [23,19]. This tripeptide motif is repeated several times in other protein components of the endocytic machinery and suggests a specific network of multivalent protein-protein interaction in endocytosis and membrane trafficking [24]. Recently, a three-dimensional structure of an NPF peptide complexed to an EH domain has been solved by NMR spectroscopy [8], which verified that the NPF residues form a $\beta$-turn that contacts the surface of the EH domain. Another example presented involved the N-terminal Src Homology 3 (SH3) domain of intersectin. When a phage-displayed combinatorial peptide library was affinity selected with this domain, the resulting peptides shared the motif Pro-Xxx-Ile/Val-Pro-Pro-Arg (PxI/VPPR), where Xxx appears to be any amino acid. A computer search of mammalian proteins in GenBank revealed a number of interesting matches, including dynamin, synaptojanin, and Son-of-sevenless (Sos). While dynamin and/or synaptojanin were already reported to interact with intersectin in pull-down [31], yeast two-hybrid [25], and co-immunoprecipitation [18] experiments, Sos was considered to be an interesting candidate interacting protein because it functions to stimulate GTP loading and activation of Ras [9]. A variety of subsequent experiments confirmed that the N-terminal SH3 domain of intersectin interacted with Sos, and that overexpression in cells of this SH3 domain could block Ras activation and downstream signaling events [1,28,29]. Thus, affinity selection of peptide ligands from phage-displayed libraries was instrumental in correctly predicting the interacting proteins of intersectin.

Once peptides that bind to another protein have been identified via phage-display, they can be used in two different avenues of drug discovery. First, the peptides can be used to validate the biological importance of a particular protein-protein interaction in the cell. For example, electroporation of peptide ligands to the SH3 domain of Lyn can block rat mast cell activation [26], and overexpression of peptide ligands to the *E. coli* Prolyl-tRNA synthetase, which were also inhibitors of charging activity *in vitro* [12], prevented bacterial growth *in vivo* [27]. Thus, peptide ligands can be used to validate particular proteins in cells as being 'good' drug targets. Second, it is possible to use the peptides in displacement assays in which libraries of natural products and small organic chemicals are rapidly screened for inhibition of particular protein-protein interactions [15,11,12]. Thus, in addition to the great utility of phage-displayed peptide ligands in mapping protein-protein interactions, they are invaluable in drug discovery efforts.

### References

1. Adams A, Thorn JM, Yamabhai M, Kay BK, O'Bryan JP. 2000. Intersectin, an adaptor protein involved in clathrin-mediated endocytosis, activates mitogenic signaling pathways. *J Biol Chem* **275**: 27414–27420.
2. Agris PF, Marchbank MT, Newman W, *et al.* 1999. Experimental models of protein–RNA interaction: isolation and analyses of tRNA(Phe) and U1 snRNA-binding peptides from bacteriophage display libraries. *J Protein Chem* **18**: 425–435.
3. Ballinger MD, Shyamala V, Forrest LD, *et al.* 1999. Semi-rational design of a potent, artificial agonist of fibroblast growth factor receptors. *Nat Biotechnol* **17**: 1199–1204.
4. Binetruy-Tournaire R, Demangel C, Malavaud B, *et al.* 2000. Identification of a *in vivo* peptide blocking vascular endothelial growth factor (VEGF)-mediated angiogenesis. *EMBO J* **19**: 1525–1533.
5. Castagnoli L, Zucconi A, Quondam M, *et al.* 2001. Alternative bacteriophage display systems. *Comb Chem High Throughput Screen* **4**: 121–133.
6. Cesareni G, Castagnoli L, Cestra G. 1999. Phage displayed

Copyright © 2001 John Wiley & Sons, Ltd. 

peptide libraries. *Comb Chem High Throughput Screen* **2**: 1–17.
7. Cwirla SE, Balasubramanian P, Duffin DJ, *et al.* 1997. Peptide agonist of the thrombopoietin receptor as potent as the natural cytokine. *Science* **276**: 1696–1699.
8. de Beer T, Hoofnagle AN, Enmon JL, *et al.* 2000. Molecular mechanism of NPF recognition by EH domains. *Nat Struct Biol* **7**: 1018–1022.
9. Downward J. 1994. The GRB2/Sem-5 adaptor protein. *FEBS Lett* **338**: 113–117.
10. Fairbrother WJ, Christinger HW, Cochran AG, *et al.* 1998. Novel peptides selected to bind vascular endothelial growth factor target the receptor-binding site. *Biochemistry* **37**: 17754–17764.
11. Grøn H, Hyde-DeRuyscher R. 2000. Peptides as tools in drug discovery. *Curr Opin in Drug Discov Dev* **3**: 636–645.
12. Hyde-DeRuyscher R, Paige LA, Christensen DJ, *et al.* 2000. Detection of small-molecule enzyme inhibitors with peptides isolated from phage-displayed combinatorial peptide libraries. *Chem Biol* **7**: 17–25.
13. Jost PJ, Harbottle RP, Knight A, *et al.* 2001. A novel peptide, THALWHT, for the targeting of human airway epithelia. *FEBS Lett* **489**: 263–269.
14. Kay BK, Kasanov J, Knight S, Kurakin A. 2000. Convergent evolution with combinatorial peptides. *FEBS Lett* **480**: 55–62.
15. Kay BK, Kurakin A, Hyde-DeRuyscher R. 1998. From peptides to drugs via phage-display. *Drug Discov Today* **3**: 370–378.
16. Li M, Yu W, Chen CH, *et al.* 1996. *In vitro* selection of peptides acting at a new site of NMDA glutamate receptors. *Nat Biotechnol* **14**: 986–991.
17. Norris JD, Paige LA, Christensen DJ, *et al.* 1999. Peptide antagonists of the human estrogen receptor. *Science* **285**: 744–746.
18. Okamoto M, Schoch S, Sudhof TC. 1999. EHSH1/Intersectin, a protein that contains EH and SH3 domains and binds to dynamin and SNAP-25. A protein connection between exocytosis and endocytosis? *J Biol Chem* **274**: 18446–18454.
19. Paoluzi S, Castagnoli L, Lauro I, *et al.* 1998. Recognition specificity of individual EH domains of mammals and yeast. *EMBO J* **17**: 6541–6550.
20. Pasqualini R, Ruoslahti E. 1996. Organ targeting *in vivo* using phage display peptide libraries. *Nature (London)* **380**: 364–366.
21. Petrenko V, Smith G. 1997. Phage Display. *Chem Rev* **97**: 391–410.
22. Rodi DJ, Makowski L. 1999. Phage–display technology–finding a needle in a vast molecular haystack. *Curr Opin Biotech* **10**: 87–93.
23. Salcini AE, Confalonieri S, Doria M, *et al.* 1997. Binding specificity and *in vivo* targets of the EH domain, a novel protein–protein interaction module. *Genes Dev* **11**: 2239–2249.
24. Santolini E, Salcini AE, Kay BK, Yamabhai M, Di Fiore PP. 1999. The EH Network. *Exp Cell Res* **253**: 186–209.
25. Sengar AS, Wang W, Bishay J, Cohen S, Egan SE. 1999. The EH and SH3 domain Ese proteins regulate endocytosis by linking to dynamin and Eps15. *EMBO J* **18**: 1159–1171.
26. Stauffer TP, Martenson CH, Rider JE, Kay BK, Meyer T. 1997. Inhibition of Lyn function in mast cell activation by SH3 domain binding peptides. *Biochemistry* **36**: 9388–9394.
27. Tao J, Wendler P, Connelly G, *et al.* 2000. Drug target validation: lethal infection blocked by inducible peptide. *Proc Natl Acad Sci U S A* **97**: 783–786.
28. Tong XK, Hussain NK, Adams AG, O'Bryan JP, McPherson PS. 2000a. Intersectin can regulate the Ras/MAP kinase pathway independent of its role in endocytosis. *J Biol Chem* **275**: 29894–29899.
29. Tong XK, Hussain NK, de Heuvel E, *et al.* 2000b. The endocytic protein intersectin is a major binding partner for the Ras exchange factor mSos1 in rat brain. *EMBO J* **19**: 1263–1271.
30. Wrighton NC, Farrell FX, Chang R, *et al.* 1996. Small peptides as potent mimetics of the protein hormone erythropoietin. *Science* **273**: 458–464.
31. Yamabhai M, Hoffman NG, Hardison NL, *et al.* 1998. Intersectin, a novel adaptor protein with two EH and five SH3 domains. *J Biol Chem* **273**: 31401–31406.

Copyright © 2001 John Wiley & Sons, Ltd. 
